# The Teeth of Children

**Published:** 1885-05

**Authors:** W. George Beers

**Affiliations:** Montreal, Canada.


AMERICAN JOURNAL

OF

DENTAL SCIENCE.

VOL. XIX. THIRD SERIES—MAY 1885. NO. I.

ARTICLE I.

### THE TEETH OF CHILDREN.

BY DR. W. GEORGE BEERS, OF MONTREAL, CANADA.

Once upon a time I thought I knew something of the predisposing causes of dental caries; but the more I read and observe, the more disposed I am to change dogmatic points of exclamation for modest marks of interrogation, and to sit as a humble hearer rather than to pose as a presumptuous preacher. Indeed, I now know that I know less than I was sure I knew when I was in my first year of practice, In respect to the increasing decay of children's teeth, I feel every day such a growing degree of ignorance that I expect soon to exclaim with the ancient philosopher in search of knowledge: "All I know is that I know nothing!" It is amazing how the omniscience of a newly-fledged dentist disappears as he gets riper experience. Dental theories in science and practice, like the toy blocks of children, seem in our day to be set up only to be upset. Thought, which one crept, now flies so fast,

> "We think our fathers wrong, so wise we grow;
> Our wiser sons no doubt will think us so."

I have a very keen sense of the risk I run in speaking here upon a subject so trite, but I venture to do so that I

may get, not that I expect to give information and because I believe that it is a subject of the gravest importance, even should it be sneeringly treated as "milk for babes." I cannot, after the open confession that I have made, expect to tell you anything new, but, as Seneca says, "a thing is never too often repeated that is not sufficiently learned." Moreover, if we all wait till we are able to contribute something purely original, many of us will never contribute anything; and if we are debarred from the dicussion of subjects familiar to us all, we may rarely discuss anything, because everything seems to have been discussed.

It is probably a perfectly safe estimate to make that in American and Canadian cities of fifty thousand people not a hundred native-born can be found between the ages of four and fourteen who have wholly escaped caries aud premature loss of some of their teeth. If this ravage was to proceed in the same ratio, men and women would be edentulous by the time they were forty, were it not for the preservative skill of the dental operator. It is a fact we must recognize that, were it not for the services daily rendered to humanity by our profession in this country, the largest proportion of the adult population would be comparatively toothless. Is it not becoming identically the same with children, so that we may say, were it not for the services daily rendered to them, the largest proportion would lose at least the deciduous molars before they were five years old, and the sixth and twelfth-year molars before they were fourteen? In fact, so common is the disease, even in Canada, that it has long been one of the popular superstitions we should aim to destroy, that the decay of children's teeth is as much to be expected as their eruption; and the children themselves have been largely educated by bitter experience to look upon it as a fatality of childhood; and may reason about it after the manner of a curious epitaph over the grave of an infant:

"Since I was so quickly done for,
I wonder what I was begun for."

Supposing it could be proved that in our public schools the lobe of one ear, or the little toe of one foot, of every tenth child were becoming tender to the touch and diminishing in size, would it not cause dismay? Would not the public mind be agitated to deep inquiry if it were found that the hair of every tenth child was turning gray, or that crow's-feet were marking their lines about their eyes? And supposing on the ear, the eyes, and the hair, at birth, there were visible indications, such as there are so often at the eruption of the teeth, which predetermined decay, would we be satisfied with mere investigation of the exciting causes, or would we not feel bound to search for causes in embryo in such a coincidence? Caries of the teeth means decay—death—as much as the graying and loss of the hair. Why should any part,—the teeth any more than the hair,—decay and die prematurely? Has it come to this, that we must accept early decay as an inevitable coincidence of their existence? Are we to believe that the teeth are the unfittest part of the body to survive, and that this trade-mark of death is impressed on the very embryo, and is to be carried in the mouths of otherwise healthy children from the time of their eruption ?

I have long since lost all suprise and wonder why the teeth of adults decay. The surprise to me is how so many escape. What boots it if the wisdom tooth should become rudimentary in civilized races? What good is it? Why should we break our hearts over the caries of the teeth of adults who have preventives, preservatives and substitutes, and think or do so little for those of children who are not able to suffer like adults, and who certainly cannot have the loss of natural teeth supplied? The only sincere surprise and pity I have left is for this caries of irresponsible children. Even the woman who expects pregnancy can make preparations against coincident or consequent dental affections. Half of the men who suffer disease and loss get just what the deserve. But it is not so with children, and I am anxious to have my gross ignorance enlightened, even at the cost of abandoning strong convictions. There must be something for mother, child, dentist, and physician to do which is not done.

The average baby is born into the world as toothless, though fortunately for its teethiug not so tough, as a turtle. Every other feature is presented in a recognizable form, pretty much as nature premeditates, whcther perfectly or imperfectly developed. One can tell fairly well if the little bit of humanity is to have its mother's eyes; or, in spite of the preternatural snub, its father's nose, or if it is to be a new departure, or an old revival of the ancestral physiognomy. Whatever physicial defect of eyes, palate, lips, or extremities is inflicted upon the child, whether congenital cataract, cleft-palate, hare-lip, or club-foot, may be detected at birth. Nature, however, loves the darkness in dealing with the teeth. She might have grown the teeth as soon as the eyes, and she might have put guards about a woman's nipples. We know the wisdom of the arrangement as it is; and one would suppose that the very invisibility in which she keeps the teeth would provoke more inquiry as to their healthy future. I know women who became physiologists when they became mothers; who went to work to study how the coming teeth were to come, with as much interest as they had ever studied the harmony of colors, or the superiority of wood or woolen for carpets. So it should be in every case. But I am not sure that parents would not learn more and care more about the teeth were they born like those of Marcus Curius, the Roman consul, who Pliny states had a full set at birth. Yet it is a fact that in many cases they are no sooner into the world than they are into trouble. Nature, as I said, forms them in the dark; and there, after birth, they lie in their secret chambers for the first five months, a hidden foe to the mother's rest. and the little owner's peace; the puzzle of the ancient physiologist, who thought the pulp a worm; the despair of the old Greek pathologist, who attributed all infantile mortality to teething, and who declared that the cause of toothache "is known only to God."

When we consider that nature is busy at work long before birth constructing the second set of teeth; that the enamel-organs of the first permanent molars appear about five months before birth, those of the second molars three months before birth, and those of the dentes sapientiæ fifteen or sixteen years before they erupt; that a child has, from its fifth year to the eruption of its first molar, forty-eight developed teeth, or the calcified germs of teeth in its jaws, and that the structural condition of both temporary and permanent is determined in embryo, we ought surely to realize the importance of study in this direction. If we accept the acid theory of caries, and especially associated with the softer character of the dentine and enamel of the deciduous teeth, we may assume that there is no more mystery as to the exciting causes in the teeth of children than in those of adults. But healthy children, from two to seven years old, have not lived enough to be exposed to the principle exciting causes of caries. Or must we declare that, just because of the softer character of their teeth, the exciting causes are more active? But, again, they are in the period of growth; nutrition is most energetic. Why is this period associated with decay? Allowing that in modern habits of life and diet we may find immediate causes, these have nothing to do with structural conditions (and it must be admitted that predisposing causes and conditions exist, for which the child is in no way responsible). I would throw the main responsibility upon the mother, given that there is no existing or hereditary disease on the part of the father; and accepting the disturbance of the nutrition of the teeth during the intra-follicular evolution as now a very common coincidence, would seek in that direction for the first, and, most often, the only predisposing causes of caries. It would seem, too, as if in certain maladies the condition of enamel is invariably disturbed, So true are the embryonic and pos-natal result of certain condition of the mother and child, that it seems to me that until we have defined and laid down in some specific way the attendant risks to which tooth-development is subject, and discover how these risks may be governed, we are siudyiug predisposing causes very much in the dark. We know that the breed of lower animals and the quality of plants and flowers may often be altered and improved. Is this to stop at mankind, or can we in any way influence the vital forces which govern the rudimentary genesis of the embryo and of the teeth? Can we do anything to secure molecular perfection; to feed germs; to prevent intra-uterine disturbance; to grow good teeth, as we can grow good geraniums? Have we any control of the embryo through the mother? Can we control nutrition,—assimilation,—or are we to abandon that idea, take the teeth as they come to us in their steady decadence, and make no effort to grow better? Is dentistry to confine its science to its practice, or is there a day to dawn when the embryologist will be consulted, as the architect or the builder; and the teeth will have become so bad that he will find large occupation in studying the idiosyncrasies and habits of people who intend to marry, or who are just married, and advising and prescribing diet, habits, etc.?

Of late, there seems to be a disposition to question the value of administering phosphates during pregnancy and the formative period of the teeth. It is important to know if the system will resolve and appropriate certain elements to different uses; to know if it will take up phosphate and carbonate of lime and magnesia, and direct them to the growing teeth. Nothing establishes truth like attacking it. It will stand if it is truth. So let us pitch into truth just to make it shine out. If what we have supposed to be truth is only fraud in disguise, the sooner we know it the better. It was a very hard pill for the old anti-amalgamites to swallow when they had been trying to believe the black coating on the old material to be an injurious preparation of mercury, instead of what it was, an innocuous preparation of silver; in other words, that they had been tryiny to believe what they wished were true. Now, unless somebody can upset better than that what many of us believe as to the value of the phosphates, by giving analytical proof from actual experiment performed upon pregnant women and the fetus at various periods, or at least upon the lower animals; unless some one can substantiate doubt by physiological and chemical proof that the formative period and the pregnant condition as regards the teeth cannot be modified by special nutrition, I venture to believe that we posess sufficient evidence to warrant the theory that lime, furnished in such forms as will be easily digested and assimilated, does contribute to dense development and perfection of tissue. Is there anything unscientific or unreasonable in the assumption tnat we can feed the germ through the blood of the mother, when we know that through the mother's blood the embryo is fed? And have we not analogous evidence in the animal and vegetable kingdoms to assure us that our creed is not founded upon guessing?

Chemical analysis tells us what it is that gives the teeth their superior hardness over the bones, and what their condition is when they are defective. It seems to be the conviction of histologists that once the enamel is formed it cannot be modified, as may be the dentine, which becomes denser by age. The structural character of enamel is, it is said, unchanged and unchangeable, except by external causes. We may not know all of the truth yet in this matter; but we know that in the development of the fetus lime is abstracted from the tissues, and especially from the teeth, of modern mothers, and that this alternation in the latter goes on in the dentine so as to predispose the unchangeable enamel to some alteration, whether structural or not. Do you not think that indirectly the enamel may be influenced through the blood of the mother, by the administration of the lime-salts which are absolutely necessary to its nutrition? It seems to me we know a good deal to encourage us. Chossat's experiments proved that by abstracting lime from food artifical softening of the bones in animals can be produced; that life will not be sustained if food is deprjved of its phosphates. We know that rickets, scrofula, and many other diseases, even difficult dentition, owe some of their origin to the deficiency of lime and magnesia. We know that, during gestation and lactation, the phosphates and carbonates are usually insufficient for the demands of the mother, and every day we see the result in the softening and decay of her teeth. At no time of life are these so urgently needed as when to lives—one in embryo—have to be supplied through the one channel. We know that if we keep lime from fowls they will have eggs without shells; that cows fed on land sown with bone phosphates will give richer milk; that wheat, planted in earth deprived of phosphates, wili die soon after its germinates; that we cannot get flowers on peas which are sown in a soil containing no phosphates. We might confine this argument entirely to facts known as to animal life and diet; and, knowing all we know, though having to admit much ignorance, may we not continue to preach to our patients the gospel of lime? Is it not a faet that the debility from which so many pregnant women suffer is due to the waste or lack of this element, and that the evidence is more than circumstantial that direct and rapid changes have been induced by its supply? If I knew enough, and had opportunity, I would like no better way of experimenting than to begin at conception, and test in a thousand cases the influence of lime upon the coming child as well as the mother. If what we know, or what we hope to know, is to be of any practical value,—and knowledge that cannot be made practical is better unknown,—we must do what we can do in the early months of pregnancy.

While we cannot, then, weigh out earthy phosphates by measure and expect them to be digested and assimilated as readily as water will be absorbed by a sponge, we can expect this from such preparations as the syrup of lacto-phosphates,* as well as the easily assimilated diets which

*One tablespoonful every day for a month, then discontinued for a month, as advised by Dr. Cushing, Chicago.

contain them. Every day the phosphates and other salts are excreted by the perspiration, the fæces and the urine, and in the latter especially there is a large excretion as a coincidence or consequence, perhaps of pregnancy. This does not imply that lime-salts are too abundant. It does not mean lack of nutrition or assimilation of the existent element, but a natural process; not an excess but a waste of matter which has become incompatible with digestion; and instead of indicating that the system has too much lime, or will not appropriate what it has, it shows a direct need for it. Blacke, of Paris, in experimenting upon the action of the phosphates, submitted a pigeon to the test of food in which they were absent. Its appetite, weight, and activity were diminished, and the fact may be noticed that it excreted more phosphates than it absorbed. The rapid loss clearly indicated the need of lime-salts, and when these were furnished they were assimilated, in spite of the quanitity being excreted, and the pigeon regained its appetite, weight and activity. In face of the evidence we possess, it would be a disastrous theory to propagate the idea that diet has no direct influence upon the origin as well as the development of the teeth. The consulting embryologist of the future will go further. He will at least do as much as the Grecians did, by keeping his patients under the impressionable influence of art, music, and sculpture. The medicines for the mind will be as much investigated as those for the body; the value of sunlight and sun-baths, of scrupulous cleanliness of body and repose of mind,—the antidotes to modern nervousness. These will be made to contribute to the growth and development of the embryo. We live in an age when the most amazing revolutions in science and discovery are received with almost perfect complacency, and it would not startle us if some modern Alphonso of Castile, who said he could have made the world better had he been consulted, would really demonstrate his ability to improve the human embryo.

I would like information upon one point. Anyone who has examined the deciduous molars previous to eruption may have found structural defects,—fissures in the grinding surfaces that look exactly like fissures found in the same teeth erupted for months. Of course, this examination must be *post mortem*, so far as the human teeth are concerned; but the investigation might be made upon dogs by vivisection. The frequency of caries in the first permanent molar, especially in the lower jaw, has been shown by Magitot, in 10,000 cases, to exceed that of any of the other teeth; while in 1,000 cases in the temporary set the same tooth in the same jaw is most frequently carious. Our lamented friend, Dr. T. B. Hitchcock, published in the *Canada Journal of Dental Science*, in 1871, a comparison of this table, prepared from his own record of 10,000 cases. While it varied in some respects, its conclusions were the same with reference to the greater frequency of decay of the lower first molars. Several theories have been proposed to account for this fact, the most popular being that the period of intra-maxillary evolution of these teeth is so prolonged and concident with a period most likely to be disturbed by diseases,—in the case of the deciduous teeth, of the mother; in the case of the permanent, of the child. With our present knowledge this seems reasonable. But if we consider the character of the superimposed gum, which for months before eruption is tense and inflamed, can it be possible that, as the result of the local irritation, the normal character of the mucus obtains an acid reaction, and that this and other acids may reach the crown of the undeveloped tooth, and slowly act upon the lime-salts of the enamel in imperfectly calcified fissures? Nasmyth's membrane would not protect the fissures, because we now know that it can be penetrated by acids. It is easy to understand why acids would thus act in the fissures of molars when it would not in the smooth surfaces of the other teeth. The condition of a child's mouth in febrile states of the body, the irritation peculiar to that part where so many teeth are growing, must vitiate the buccal fluids. I was surprised, in testing the saliva of nursing children before the eruption of the teeth, to find a decided acid reaction in every case. Infants do swallow every particle taken into the mouth. The cheeks, the maxillæ folds, the tongue, retain portions of soft food, and these sour and become acid. One of the national customs among the French Canadian peasantry is to put a small cloth bag of bread and sugar, soaked in milk, into the mouths of infants to keep them quiet. Frequently the result is to cause vomiting and an excess of acid in the stomach. I doubt if it is generally realized to what extent acids are present in the mouth before the eruption of the teeth. Now, I venture to believe that just as easily, if not more so, as iodine and aconite painted on the gums can reach and act upon the periosteum, these acids can reach and act upon the crowns of the undeveloped molars.

As I propose in a future paper, with your permission to discuss the specific result of the diseases of pregnancy and infancy in their effects upon the teeth, I will not allude to the subject here. To my mind, neither physicians nor dentists will ever do all that could or should be done. It remains with the mothers to learn more about the origin and development of the teeth, and to take as much interest in the embryology of their future offspring as they do in house-plants which they grow from the seed, or as some do in the breeding of pug dogs.

The care of children's teeth after eruption ought to occupy more attention. It falls naturally upon the mother; but it ought to occupy as much of our thought as treatment. Every one of has seen hundreds of disheartening cases; children of eleven and twelve years having twenty or more carious cavities in the teeth,—that discouraging decay between the lower incisors which marks caries of embryonal origin, It is remarkable how the large proportion of these cases can be traced back to disturbances and diseases during pregnancy, or in the early months and years after birth.

One of the best means to make parent and child value the teeth which should last forever is to make them realize the importance of those which should last for six or seven years, during which time the child is entirely dependent upon them for mastication. Every mother ought to know the process by which the deciduous teeth are removed by nature. My experience is that most parents think they are shed as a canary gets rid of its feathers, or a deer its antlers; Some imagine that they are lost as a crab looses its shell or that it is a process of moulting to be continued at intervals during growth, like that of the lobster. People are prepared to believe anything about anything of which they know nothing, and they ought to be surprised by being taught the truth.

The popular superstition that the deciduous teeth are only of temporary importance, and that their premature loss is only one of appearance, not of function, like all superstitions, was founded upon ignorance, and is receiving its death-blow in this country. But, as a rule, the first eight or twelve years of a child's life is too often a time of neglect, so far as the teeth are concerned; and if there is any time when every tissue and organ should have the utmost care, it is when they are in rapid growth, when vitality depends upon what goes into the mouth, and from the mouth into the stomach. As a rule, children are never taught the object of their teeth and the need for their exercise. Example in this fast age of quick eating is rarely given, for it may be said of most of us, as Plato said of the citizens of Agrigentum, "They eat as if they had not an hour to live." Mere eating is not mastication. Let this be the first idea of the function of the deciduous as well as of the permanent teeth,—That they are intended as human mills to prepare the food for digestion, and that as machinery rusts out quicker than it wears out, so teeth which are not exercised by mastication are more predisposed to decay than those which have plenty of hard food to grind. The lessons of hygiene are so simple that they are rarely observed, just because of their simplicity,—daily use of the badger-hair tooth-brush, percipitated chalk, and castile soap. Mothers ought to have mouth-mirrors, and ought to examine the child's teeth every month of every year. I have seen children who have looked forward to the eruption of a tooth as a happy event in their lives. Why should not this be a life-lesson of pleasure to a child from the time it has intelligence enough to watch for its dental development? It is taught to take care of its hair, its skin, its nails, its toes, its eyes. A child's deciduous teeth are as much a work of the Creator and as important for the time being as the health of its hair, its skin, its nails, its toes, or even its eyes. If nature made any mistake, however, it was in giving us so many teeth that they are not only undervalued, but so easily replaced. If a child had twenty eyes and only two teeth, the custom which governs so many parents in the care of them would be reversed, especially if the twenty eyes were temporary, to be replaced by thirty-two successors, and the two teeth had to be carried to the grave. If we cannot grow better teeth for children, we must do more than we have yet done to keep the poor ones they have. Even in this direction, I feel we will never be able to do the best for their teeth until the great discovery comes with the permanent translucent soft filling. Children, even of an older growth, will then have one of the miseries of life ameliorated, and the structural poverty we regret will be met by some after compensation that will do much to lessen the need for prosthetic dentistry.

I should have concluded long ago, but I must do so now, and with reiterating my conviction of my own ignorance. Confucius has a fine thought: "What you know, to know that you know it, and what you do not know, to allow that you do not know it,—that is knowledge." I have tried to show you how little I know that I may the sooner know more. No doubt you will assure me that in this I have splendidly succeeded.—*New York Odontological Society roceedings.*

